# Heterojunction Incorporating Perovskite and Microporous Metal–Organic Framework Nanocrystals for Efficient and Stable Solar Cells

**DOI:** 10.1007/s40820-020-00417-1

**Published:** 2020-03-28

**Authors:** Xuesong Zhou, Lele Qiu, Ruiqing Fan, Jian Zhang, Sue Hao, Yulin Yang

**Affiliations:** MIIT Key Laboratory of Critical Materials Technology for New Energy Conversion and Storage, School of Chemistry and Chemical Engineering, Harbin Institute of Technology, Harbin, 150001 People’s Republic of China

**Keywords:** Metal–organic framework, Nanocrystal, Heterojunction, Light-harvesting layer, Perovskite solar cell

## Abstract

In this paper, we present a facile approach to enhance the efficiency and stability of perovskite solar cells (PSCs) by incorporating perovskite with microporous indium-based metal–organic framework [In_12_O(OH)_16_(H_2_O)_5_(btc)_6_]_n_ (In-BTC) nanocrystals and forming heterojunction light-harvesting layer. The interconnected micropores and terminal oxygen sites of In-BTC allow the preferential crystallization of perovskite inside the regular cavities, endowing the derived films with improved morphology/crystallinity and reduced grain boundaries/defects. Consequently, the In-BTC-modified PSC yields enhanced fill factor of 0.79 and power conversion efficiency (PCE) of 20.87%, surpassing the pristine device (0.76 and 19.52%, respectively). More importantly, over 80% of the original PCE is retained after 12 days of exposure to ambient environment (25 °C and relative humidity of ~ 65%) without encapsulation, while only about 35% is left to the pristine device.

**Electronic supplementary material:**

The online version of this article (10.1007/s40820-020-00417-1) contains supplementary material, which is available to authorized users.

## Highlights

- Microporous indium-based metal–organic framework (In-BTC) nanocrystals are synthesized under an extremely mild condition.
- Perovskite/In-BTC heterojunction films possess improved morphology/crystallinity and reduced grain boundaries/defects.
- In-BTC-modified perovskite solar cells exhibit significantly enhanced efficiency of 20.87% and long-term stability.

**Electronic supplementary material:**

The online version of this article (10.1007/s40820-020-00417-1) contains supplementary material, which is available to authorized users.

## Graphical abstract

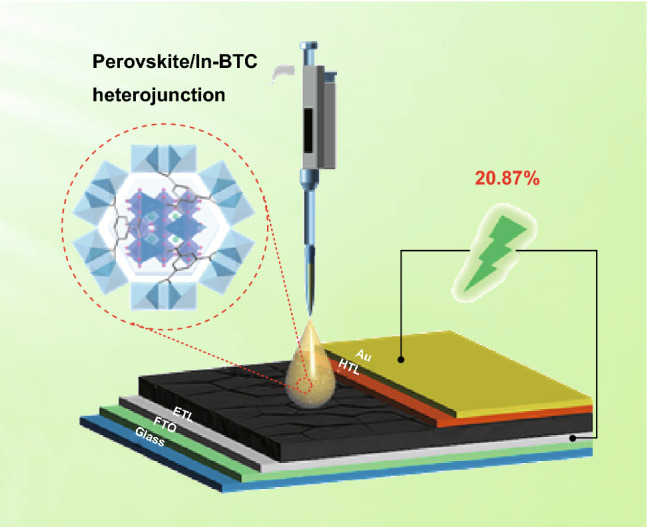

## Introduction

Since it first debuted as a photosensitizer in 2009 [1], organic–inorganic hybrid perovskite has been proved to be an excellent light-harvesting material, owing to the advantages of wide absorption band, ambipolar charge transporting, long carrier diffusion length, and tunable bandgaps [2–5]. On this basis, perovskite solar cells (PSCs) have attracted wide attention and achieved incredibly rapid development [6–9]. The power conversion efficiency (PCE) of devices dramatically increases from 3.8 to 25.2% within only a few years [10–12]. Nevertheless, the poor long-term stability of PSCs, mainly related to the intrinsic defects inside perovskite films, is still one of the biggest stumbling blocks on the route to their commercialization [13–17]. Many evidences have been pointed out that the perovskite films possess polycrystalline nature and generally suffer from the threat of moisture, oxygen, heat, and UV radiation, due to the unavoidable formation of a large numbers of grain boundaries and defects under the solution-phase fabrication processes [18–22]. To address these issues, various strategies have been adopted to enhance the robustness of perovskite films against the external stresses, such as composition modulation or introducing functional interlayers/additives [23–28].

Recently, the nanoscale metal–organic frameworks (MOFs) have drawn increasing attention in the photovoltaic field [29–32]. Specifically, the advantages of excellent stability, interconnected porosity, high specific surface area, and decent solution processability endow nano-MOF materials with promising potential as the functional interlayers or additives [33, 34]. Moreover, the photovoltaic properties of MOFs could be easily manipulated by tuning the combination of metal ions/clusters and organic linkers, to meet the practical demands of corresponding devices [35]. In spite of this, the introduction of MOFs into PSCs is still rarely reported, especially for the investigations involving perovskite/MOF heterojunctions. In 2015, Chang et al. [36] first added microporous MOF-525-Zr(IV) nanocrystals into the perovskite precursor solution. The resulting perovskite thin films with improved morphology and crystallinity have delivered a PCE of 12.0%. Very recently, Lee et al. [37] mixed another two nanoscale Zr(IV)-based MOFs (UiO-66 and MOF-808) with the perovskite precursor. The corresponding PSCs assumed high PCE up to 18.01% and 17.81%, respectively. Meanwhile, it has been proved that the devices fabricated by perovskite/UiO-66 or perovskite/MOF-808 heterojunctions could realize better photovoltaic performance than those employing the MOFs as interlayers. More importantly, the UiO-66/MOF-808-hybrid PSCs possessed significantly enhanced long-term stability over the pristine devices. Obviously, employing a perovskite/MOF heterojunction as the light-harvesting layer is indeed an effective strategy to improve the efficiency and stability of PSCs. Nevertheless, to the best of our knowledge, the above-mentioned are the total examples of perovskite/MOF heterojunctions used in PSCs, and the investigations on their effectiveness for overall devices are still insufficient. In this case, incorporating other MOF nanocrystals with robust framework and suitable pore size into the perovskite films is considered of great significance to expand the application of such materials in photovoltaic field.

**Fig. 1:**
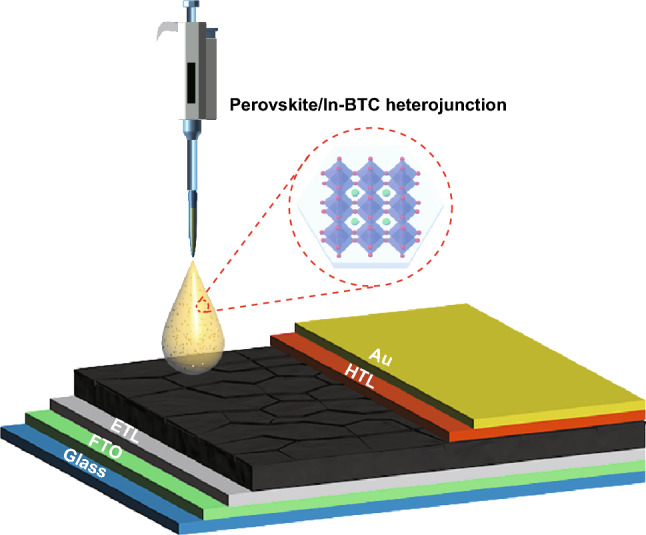
Schematic diagram for fabricating n-i-p PSCs with the perovskite/In-BTC heterojunction as light-harvesting layer

Generally, Zr-based MOF materials require intricately modulated synthesis under a high temperature, which brings unavoidable difficulty to the preparation process of samples [38–41]. In contrast, the In(III)-based MOFs with relatively flexible synthesis conditions and adequate stability seem to be potential candidates for effective perovskite/MOF heterojunctions. Herein, a stable microporous In(III)-based MOF, named [In_12_O(OH)_16_(H_2_O)_5_(btc)_6_]_n_ (In-BTC), was synthesized by a simple self-assembly strategy under extremely mild condition (85 °C, 6 h, and water/acetonitrile mixture). In particular, the particle size of as-synthesized samples could be easily adjusted from the micron- to nanoscale by tuning the volume ratio of water and acetonitrile. Choosing the nanoscale In-BTC crystals as additive, we systematically investigated the relationship between the concentrations of In-BTC in perovskite precursor solution and the photovoltaic performance of corresponding perovskite/In-BTC heterojunction-based PSCs. At an In-BTC adding amount of 2.0 mg mL^−1^, the heterojunction films with obviously improved morphology and crystallinity were obtained. Naturally, the interfacial electrical contact, photoresponse, and robustness against the ambient environment of derived PSCs could be significantly enhanced. As a result, the optimal In-BTC-modified device realized a PCE of 20.87%, which is superior to the pristine one (19.52%). More importantly, the perovskite/In-BTC heterojunction-based PSCs could maintain over 80% of the initial PCE after being stored in ambient environment (25 °C and relative humidity of ~ 65%) for 12 days without encapsulation, in contrast to the rapid decay for pristine devices. Figure 1 presents the schematic diagram for fabricating n-i-p PSCs with the perovskite/In-BTC heterojunction as light-harvesting layer.

## Experimental Section

### Preparation of [In_12_O(OH)_16_(H_2_O)_5_(btc)_6_]_n_

In this work, we report a simple approach to self-assembly synthesize monodisperse microporous [In_12_O(OH)_16_(H_2_O)_5_(btc)_6_]_n_ (In-BTC) nanocrystals for the first time. A well-stirred mixture of l,3,5-benzenetricarboxylic acid (H_3_btc, 0.10 mmol) and indium nitrate pentahydrate (In(NO_3_)_3_·5H_2_O, 0.20 mmol) in water/acetonitrile was sealed in the 23-mL Teflon-lined stainless-steel autoclave and heated at 85 °C for 6 h. After cooling to the room temperature, the particles were washed with ethanol and deionized water, followed by the centrifugation at 10,000 rpm and ultrasonication for the redispersion in ethanol. Particles as hexagonal prism slices with different well-defined sizes were collected, depending on the volume ratio of water and acetonitrile in reaction system (9:1 for ~ 5 μm, 8:2 for ~ 1 μm, and 7:3 for ~ 150 nm, respectively). Finally, the particles were dried at 150 °C for 12 h and stored in a sealed bottle for further use.

### Fabrication of n-i-p PSCs

Fluorine-doped tin oxide (FTO) glass (14 Ω m^−2^ square, NSG, Japan) substrates were partially etched by zinc power and hydrochloric acid solution (HCl, 4 mol L^−1^) and ultrasonically washed with deionized water, acetone, and ethanol for 30 min, respectively. Then, the substrates were blown to dry by nitrogen and treated with UV ozone for 15 min. The compact titanium dioxide (c-TiO_2_) films were prepared by mixing titanium (IV) isopropanol (175 μL) and HCl (17.5 μL, 3 mol L^−1^) in 1.25 mL isopropanol, spin-coating at 3000 rpm for 30 s, and sintering at 500 °C for 30 min. After cooling, PC_61_BM (5 mg mL^−1^) was spin-coated onto the c-TiO_2_ films at 3000 rpm for 30 s and heated at 70 °C for 10 min. Subsequently, the perovskite films were formed by further spin-coating a Cs_0.05_FA_0.81_MA_0.14_PbI_2.55_Br_0.45_ (1.2 mol L^−1^) precursor solution, which was prepared by mixing CsI, FAI, MABr, PbI_2_, and PbBr_2_ at 60 °C according to the molar ratio. For the perovskite/In-BTC heterojunction films, different quantities of In-BTC nanocrystals were added into the above pristine perovskite precursor solution to form stable suspension with different concentrations of In-BTC for further spin-coating. Afterward, the substrates were heated at 100 °C for 15 min. The hole transport layer (HTL) films (72.3 mg of Spiro-OMeTAD in 1 mL chlorobenzene with the addition of 17.5 μL of Li-TFSI solution (520 mg in 1 mL acetonitrile), and 28.8 μL of *t*-BP) were spin-coated above the perovskite films at 3000 rpm for 30 s. Lastly, Au was sublimated on the top of HTL films, serving as a metal electrode.

### Measurements and Characterizations

The scanning electron microscope (SEM) images were taken by Rili SU 8000HSD Series Hitachi New Generation Cold Field Emission SEM. The powder X-ray diffraction (PXRD) patterns were recorded in the 2*θ* range of 5–50° using Cu Kα (*λ* = 1.5418 Å) radiation with a Shimadzu XRD-6000 X-ray diffractometer. The nitrogen (N_2_) adsorption–desorption isotherm was measured on Micromeritics ASAP 2020. The thermal gravimetric analysis (TGA) was performed on a ZRY-2P thermogravimetric analyzer from 30 to 500 °C with a heating rate of 2 °C min^−1^ under a flow of air. The UV–Vis absorption spectra were recorded by a Shimadzu and SPECORD S600 spectrophotometer. The photoluminescence (PL) performance was investigated by the steady-state fluorescence spectrometer FLS920. The cyclic voltammetry (CV) was obtained using the Gamry electrochemical workstation with Pt plate as working electron, Pt slice as counter electrode, and Ag/AgCl electrode as reference electrode in tetrabutylammonium hexafluorophosphate (Bu_4_NPF_6_, 0.1 mol L^−1^) chlorobenzene solutions at a scan rate of 50 mV s^−1^. The photocurrent density–voltage (*J*–*V*) characteristics were recorded by a Gamry interface 1000E with a simulated light intensity of 100 mW cm^−2^ and a scanning rate of 100 mV s^−1^. The active area of 0.06 cm^2^ was confirmed by the aperture shade mask. The IPCE spectra were collected using a Newport IPCE measurement system, and the light intensity was corrected by silicon detector (model no. 71675, Newport, USA). The X-ray photoelectron spectroscopy (XPS) spectra were measured at room temperature using an ESCALAB-250 spectrometer (Thermo, America) diffraction instrument.

## Results and Discussion

### Characterization of In-BTC

**Fig. 2:**
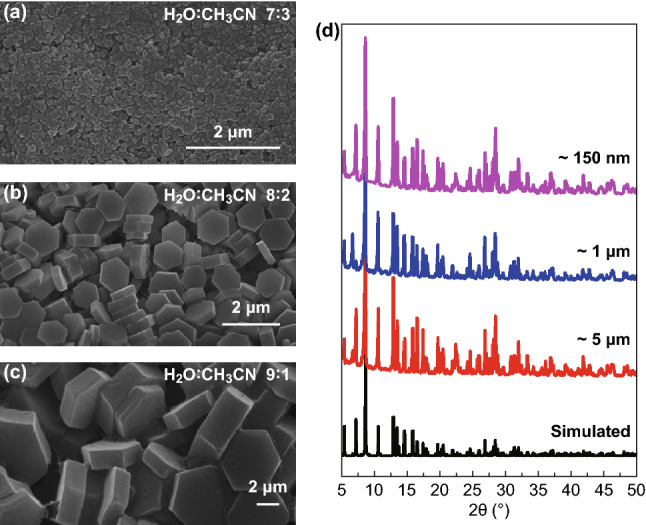
SEM images of as-synthesized In-BTC samples with different particle sizes: **a** ~ 150 nm, **b** ~ 1 μm, and **c** ~ 5 μm. **d** The corresponding PXRD patterns

The In-BTC samples were self-assembly synthesized under a extremely mild condition (85 °C, 6 h, and water/acetonitrile mixture) in the presence of indium nitrate pentahydrate (In(NO_3_)_3_·5H_2_O) and l,3,5-benzenetricarboxylic acid (H_3_btc). Interestingly, the particle size of as-synthesized In-BTC samples could be easily controlled by adjusting the volume ratio of water and acetonitrile in reaction system (9:1 for ~ 5 μm, 8:2 for ~ 1 μm, and 7:3 for ~ 150 nm, respectively). As shown in Fig. 2a–c, the unaltered hexagonal prism slice morphology is clearly visualized by corresponding SEM images. Figure 2d shows the PXRD patterns of as-synthesized In-BTC samples with different particle sizes. All the PXRD patterns match well with the simulation, indicating the high phase purity of products.

The In-BTC possesses a three-dimensional (3D) framework, which consists of isolated trinuclear units connected with infinite 2D layers of indium-centered octahedrons through the [btc]^3−^ ligands (Fig. S1). For the sake of analysis, three types of crystallographically inequivalent indium ions are labeled as In1, In2, and In3, respectively. The trinuclear unit contains three In1-centered octahedrons sharing one *μ*_3_-oxo (Fig. S2a), while the 18-membered hexagonal ring-based infinite 2D layer on *ab* plane derives from the bridging between two types of indium ions (In2 and In3) through *µ*_2_-OH with a *cis*–*cis*–*trans* corner-sharing sequence (Fig. S2b–e). The connection of *μ*_3_-oxo-centered trinuclear units with infinite 2D hexagonal fragment through [btc]^3−^ ligands not only ensures the robustness of overall structure but also forms three kinds of interconnected cavities (Fig. S3).

**Fig. 3:**
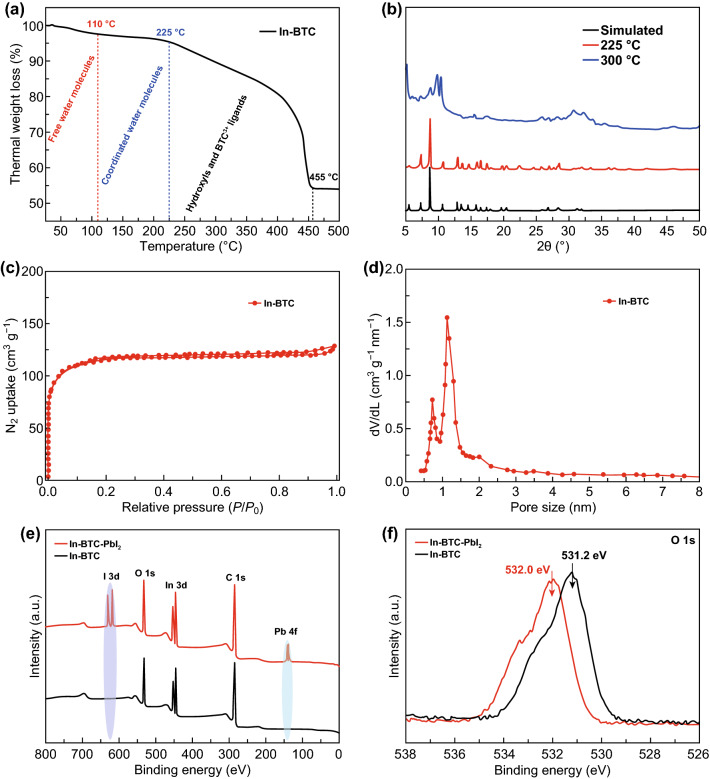
**a** TGA curve of In-BTC nanocrystals under air atmosphere (2 °C min^−1^). **b** PXRD patterns of In-BTC nanocrystals after thermal treatment at different temperatures. **c** Nitrogen adsorption–desorption isotherm and **d** pore size distribution of the In-BTC nanocrystals. XPS **e** full spectra and **f** O 1 s spectra of the pure In-BTC or the mixture of In-BTC and PbI_2_

In order to evaluate the stability of In-BTC nanocrystals, the thermal gravimetric analysis (TGA) was carried out under the air atmosphere in a temperature range from 30 to 500 °C with a heating rate of 2 °C min^−1^. As shown in Fig. 3a, the first step of weight loss between the room temperature and 110 °C corresponds to the release of free water molecules trapped in the cavities of In-BTC framework. The second slope that stops at around 225 °C should be attributed to the removal of coordinated water molecules. After 455 °C, the overall framework of In-BTC completely collapses and converts into indium oxide (In_2_O_3_). The PXRD patterns of as-synthesized In-BTC nanoparticles after thermal treatment at different temperatures for 12 h have further proved the structural stability below 225 °C (Fig. 3b), which is adequate for its application in PSCs.

Subsequently, the pore properties of as-synthesized In-BTC nanocrystals were tested by the nitrogen (N_2_) adsorption–desorption measurement. Figure 3c displays its typical I isotherm, which corresponds to the microporous adsorption process. Meanwhile, the Brunauer–Emmett–Teller (BET) specific surface area was estimated to be 331.62 m^2^ g^−1^, and the obtained two pore sizes (~ 0.7 and ~ 1.1 nm, Fig. 3d) are in excellent agreement with the cavity sizes visualized in the single-crystal structure analysis of In-BTC (Fig. S3). The interconnected micropores are considered to allow the penetration of precursor solution, enhancing the miscibility/compatibility between In-BTC nanocrystals and perovskite. Moreover, the polar sites of In-BTC might temporarily coordinate with Pb^2+^ ions to induce the preferentially nucleation of perovskite inside the regular cavities, and synchronously modulate the crystallization rate during the film evolution process [42]. In this regard, the XPS spectrum of In-BTC nanocrystals in the presence of PbI_2_ was measured (Fig. 3e, f). The electron binding energy of O 1 s for pure In-BTC samples is obviously enhanced from 531.2 to 532.0 eV after mixing with PbI_2_, which could be attributed to the coordination interaction between terminal oxygen sites and Pb^2+^ ions. According to the above-mentioned, a dense thin film with improved morphology/crystallinity and reduced grain boundaries/defects could be realized by introducing In-BTC nanocrystals into the perovskite precursor solution.

**Fig. 4:**
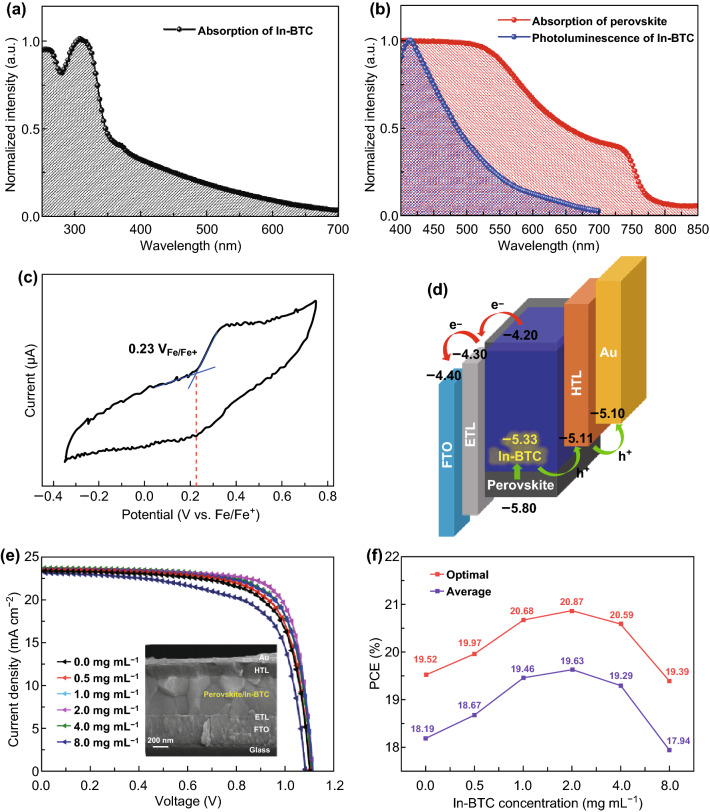
**a** UV–Vis absorption spectrum of In-BTC nanocrystals. **b** PL spectrum of In-BTC nanocrystals and the UV–Vis absorption spectrum of perovskite film. **c** CV curve of In-BTC nanocrystals. **d** Energy-level diagram of PSCs with perovskite/In-BTC heterojunction as the light-harvesting layers. **e** *J*–*V* curves for PSCs employing perovskite/In-BTC heterojunction with different addition concentrations of In-BTC nanocrystals. The inset shows the cross-view SEM image of PSCs. **f** Optimal and average PCE values obtained from 20 separated PSC devices

The UV radiation has been proved to be harmful to the stability of perovskite films in recent researches [43]. The intense absorption of In-BTC nanocrystals in ultraviolet region suggests that the high-energy photons could be effectively filtered after introducing In-BTC into the perovskite intrinsic layer as an additive (Fig. 4a). In addition, the photoluminescence (PL) spectrum of In-BTC samples was further measured (Fig. 4b). The maximum PL emission peak is centered at 415 nm, indicating that the In-BTC nanocrystals could also provide a down-conversion of the UV radiation [44, 45]. Meanwhile, inferring from the high overlap between the PL emission spectrum of In-BTC samples and the UV–Vis absorption spectrum of perovskite films, there should be a Förster resonance energy transfer effect. Therefore, it is of great benefits to enhance the photoresponse of corresponding PSCs by utilizing perovskite/In-BTC heterojunction as the light-harvesting layer.

As shown in Fig. 4c, the electrochemical redox potential of In-BTC nanocrystals has been determined to be 0.23 V versus Fc/Fc^+^ (ferrocene/ferrocene^+^) via cyclic voltammetry (CV). According to the relation of *E*_HOMO_ = − (*E*_[onset, ox vs. Fc/Fc+]_ + 5.1) (eV), the highest occupied molecular orbital energy level (*E*_HOMO_) of In-BTC is estimated to be − 5.33 eV, which is in good agreement with the result (− 5.41 eV) obtained from the ultraviolet photoelectron spectroscopy (UPS) (Fig. S4). Obviously, the *E*_HOMO_ of In-BTC is located between those of the perovskite (− 5.80 eV) and hole transport material (Spiro-OMeTAD: − 5.11 eV) [46]. The highly matched *E*_HOMO_ values indicate that the In-BTC nanocrystals could facilitate the extraction of hole carriers from perovskite intrinsic layers to hole transport layers (HTLs) (Fig. 4d), further suggesting the feasibility of perovskite/In-BTC heterojunction in PSCs.

### Device Performance of Perovskite/In-BTC Heterojunction-Based n-i-p PSCs

To determine the optimal composition of perovskite/In-BTC heterojunction, five different addition concentrations (0.5, 1.0, 2.0, 4.0, and 8.0 mg mL^−1^, respectively) of In-BTC nanocrystals in perovskite precursor solution were selected for fabricating PSCs and comparing with the pristine devices (0.0 mg mL^−1^). The photovoltaic performance of corresponding PSCs is recorded in Table 1, Fig. S5, and Fig. 4e, f. (The inset shows the cross-view SEM image of a complete device with typical n-i-p configuration of fluorine-doped tin oxide (FTO)/compact TiO_2_ (c-TiO_2_)/PC_61_BM/Cs_0.1_FA_0.747_MA_0.153_PbI_2.49_Br_0.51_/In-BTC/HTL/Au.) It can be observed that the optimal PCE of 20.87% was achieved at the In-BTC addition concentration of 2.0 mg mL^−1^. The corresponding open-circuit voltage (*V*_oc_), short-circuit current density (*J*_sc_), and fill factor (FF) values were 1.12 V, 23.55 mA cm^−2^, and 0.79, respectively. Such photovoltaic performance is significantly superior to that of the pristine one without In-BTC additive (PCE = 19.52%, *V*_oc_ = 1.11 V, *J*_sc_ = 23.14 mA cm^−2^, and *FF* = 0.76, respectively). Moreover, the perovskite/In-BTC heterojunction-based PSCs (2.0 mg mL^−1^) exert a higher average PCE (19.63%, estimated from 20 separated devices) than the pristine devices (18.19%), verifying a great reproducibility. Subsequently, the steady-state *J*_SC_ and corresponding PCE at the maximum power point for various PSCs were measured to confirm the accuracy of PCE values obtained from the *J*–*V* curves. As shown in Fig. S6a, b, the In-BTC-modified PSC possesses a PCE value of 20.66% with a steady-state *J*_SC_ of 21.28 mA cm^−2^, while the results for pristine device are 19.15% and 20.17 mA cm^−2^, respectively, which are all extremely close to those obtained from the *J*–*V* curves. In addition, the reliability of *J*_SC_ was also ensured by the incident photon-to-electron conversion efficiency (IPCE) results of corresponding PSCs. As shown in Fig. S6c, the integrated *J*_SC_ of 22.85 and 23.40 mA cm^−2^ for the pristine and In-BTC-modified devices, respectively, is in good agreement with the results of *J*–*V* measurements as well. Notably, compared to pristine device, the In-BTC-modified PSC possesses obviously enhanced photoresponse from 300 to 550 nm. This should be attributed to the Förster resonance energy transfer between In-BTC and perovskite, since the PL emission spectrum of In-BTC and the UV–Vis absorption spectrum of perovskite show a high overlap in this spectral region, as discussed above.

**Table 1:** Photovoltaic performance of the PSCs employing perovskite/In-BTC heterojunction with different addition concentrations of In-BTC nanocrystals

Concentrations of In-BTC nanocrystals	*V*_*oc*_ (V)	*J*_*sc*_ (mA cm^−2^)	FF	PCE (%)
0.0 mg mL^−1^	1.08 ± 0.04	22.65 ± 0.87	0.75 ± 0.03	18.19 ± 1.33
0.5 mg mL^−1^	1.09 ± 0.03	22.86 ± 0.78	0.75 ± 0.03	18.77 ± 1.30
1.0 mg mL^−1^	1.10 ± 0.03	22.95 ± 0.86	0.76 ± 0.04	19.46 ± 1.22
2.0 mg mL^−1^	1.10 ± 0.02	22.99 ± 0.79	0.77 ± 0.03	19.63 ± 1.24
4.0 mg mL^−1^	1.09 ± 0.03	22.82 ± 0.80	0.77 ± 0.03	19.29 ± 1.30
8.0 mg mL^−1^	1.07 ± 0.03	22.54 ± 0.82	0.74 ± 0.03	17.94 ± 1.45

All error bars were estimated from 20 separated devices

To investigate the charge dynamics at the interfaces of light-harvesting layer for corresponding PSCs, the transient photocurrent and photovoltage decay curves were recorded in Fig. S7a, b, respectively. The In-BTC-modified device shows a faster photocurrent decay (1.85 μs) than the pristine one (2.66 μs), indicating the efficient interfacial carrier migration, which might be related to the highly matched energy level of In-BTC with perovskite and hole transport material. Meanwhile, the delayed photovoltage decay from 3.18 μs for the pristine device to 4.57 μs for the In-BTC-modified one indicates that employing In-BTC nanocrystals as an additive can realize a longer carrier lifetime for PSCs. This can be explained by the defect passivation effect of In-BTC on perovskite crystal films, effectively suppressing the recombination of photogenerated carriers. Furthermore, the electrical impedance spectroscopy (EIS) measurements were performed for the PSCs with pure perovskite or perovskite/In-BTC heterojunction (2.0 mg mL^−1^) via Gamry electrochemical workstation under AM 1.5G illumination with 0 bias voltage at frequency of 0.1 Hz to 105 Hz, to gain more insights into the photogenerated carrier migration and recombination behavior of devices. As shown in Fig. S8, two semicircles could be observed from the Nyquist plots, where the left small one in high-frequency region is related to the charge transfer resistance (*R*_tr_) and the other one on the right in low-frequency region corresponds to the recombination resistance (*R*_rec_), respectively. Significantly, compared to the pristine PSC, the In-BTC-modified device possesses reduced *R*_tr_ (from 906.2 to 743.1 O) and increased *R*_rec_ (from 1922.4 to 2985.7 O). This indicates that employing perovskite/In-BTC heterojunction could effectively improve the photogenerated carrier migration and extraction and concurrently limit the recombination, enhancing the device performance of PSCs.

The defects inside perovskite films are a major inducement of the hysteresis phenomenon, which leads to an inaccurate estimation of the device performance. To investigate the hysteresis behavior of corresponding PSCs, the *J*–*V* curves were, respectively, measured in both forward and reverse scanning directions (Fig. S7c, d). Obviously, the perovskite/In-BTC heterojunction-based device possesses a lower hysteresis index (HI) of 0.11 than the pristine one (0.14), further proving that the In-BTC nanocrystals have made efforts in the defect passivation of perovskite films.

**Fig. 5:**
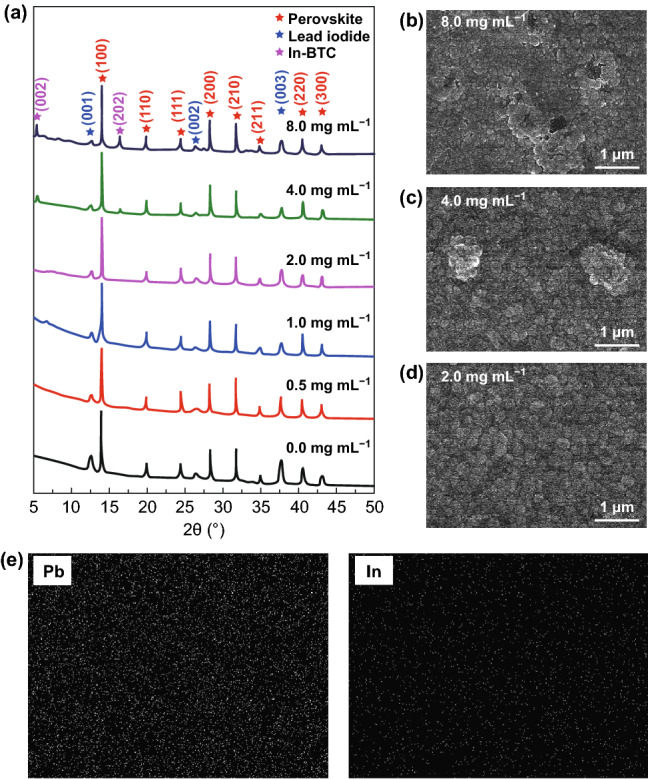
**a** PXRD patterns of pristine perovskite thin films and perovskite/In-BTC heterojunction films with different addition concentrations of In-BTC nanocrystals. **b**–**d** SEM images of corresponding films. **e** SEM–EDS images of optimal perovskite/In-BTC heterojunction film (2.0 mg mL^−1^)

In addition, the difference in photovoltaic performance, especially for *FF* of the In-BTC-modified PSCs with various In-BTC addition concentrations, leads us to further investigate the properties of corresponding perovskite/In-BTC heterojunction films. Figure 5a shows the PXRD patterns, and the diffraction peaks of pristine perovskite thin film at 14.02°, 19.81°, 24.50°, 28.46°, 31.82°, 35.10°, 40.71°, and 43.14° can be correctly indexed as the (100), (110), (111), (200), (210), (211), (220), and (300) planes of perovskite crystal structure, respectively, while the remaining three peaks at 12.57°, 26.45°, and 37.66° belong to the (001), (002), and (003) planes of excessive PbI_2_, respectively. Notably, with the increase in In-BTC addition concentrations, the intensity ratio of perovskite (100) peak and PbI_2_ (001) peak is significantly enhanced. This result indicates that the In-BTC nanocrystals are beneficial to improve the crystallinity of derived perovskite films. Moreover, the top-view SEM images of corresponding films are presented in Figs. 5b–d and S9. It can be clearly observed that the crystalline grain size of perovskite/In-BTC films is obviously larger than that of the pristine one, further verifying the positive effects of In-BTC nanocrystals on improving the crystallinity of derived perovskite films. Meanwhile, the overall morphology of thin films is also optimized to some extent. In other words, the grain boundaries and defects of the perovskite films have been effectively reduced by introducing In-BTC nanocrystals, and the photogenerated carrier migration inside the films as well as the electrical contact at associated interfaces could be accordingly improved. Nevertheless, the excessive addition of In-BTC nanocrystals (4.0 or 8.0 mg mL^−1^) would lead to the phase separation of perovskite and In-BTC, which can be visualized as the malignant changes in film morphology, as shown in Fig. 5b, c. Meanwhile, two new diffraction peaks at 5.37° and 16.45° appear in the corresponding PXRD patterns, which belong to the (002) and (202) planes of In-BTC, respectively. The SEM–EDS images show the element distribution of optimal perovskite/In-BTC heterojunction films (2.0 mg mL^−1^) (Figs. 5e and S10). The homogeneous distributions of these elements benefit from the nanoscale particles and interconnected micropores of In-BTC additive.

As shown in Fig. S11a, the UV–Vis absorption spectra of perovskite/In-BTC heterojunction films with different In-BTC addition concentrations were also measured. Clearly, the absorbance of corresponding films exhibits an upward trend from 400 to 550 nm with the increase in In-BTC addition concentrations, suggesting the positive role of In-BTC additive. The enhanced absorption portends that more photogenerated carriers could be excited. To confirm this point, the PL spectra of the above heterojunction films were further measured, as shown in Fig. S11b. Consistently, the PL intensity also exerts an upward trend with the increase in In-BTC additive amounts. Meanwhile, the redshifts of PL emission peaks can be observed, which should be related to the interaction between In-BTC nanocrystals and perovskite. In addition, the time-resolved photoluminescence (TRPL) measurement was carried out to investigate the charge dynamics of various films. As shown in Fig. S12, all of the perovskite/In-BTC heterojunction films show slower PL decay than the pristine one, indicating that In-BTC nanocrystals could effectively restrain the carrier recombination. It is worth mentioning that although a relatively high In-BTC addition concentration (4.0 or 8.0 mg mL^−1^) has further enhanced the spectral properties of derived perovskite/In-BTC heterojunction films, the low photovoltaic performance of corresponding PSCs was obtained, probably resulting from the poor film morphology.

The significantly enhanced photovoltaic performance of optimal perovskite/In-BTC heterojunction-based device (2.0 mg mL^−1^) encourages us to further investigate its long-term stability. As shown in Fig. S13, the corresponding PCE values were recorded following with the different storage times in ambient environment at room temperature and a relative humidity (RH) of ~ 65% without encapsulation. After 12 days, 81.3% of initial PCE was maintained for the In-BTC-modified solar cell benefitting from the improved morphology/crystallinity and reduced grain boundaries/defects of heterojunction film, while that of the pristine device dropped down to 35.4%. This result clearly reflects the effectiveness of perovskite/In-BTC heterojunction in enhancing the stability of PSCs against high-humidity environment.

## Conclusions

In summary, we purposefully synthesized microporous In-BTC nanocrystals and systematically described the effectiveness of perovskite/In-BTC heterojunction as the light-harvesting layers in realizing high-efficiency and long-term stable PSCs. The In-BTC additive was shown to improve the morphology and crystallinity and simultaneously reduce the grain boundaries and defects of perovskite films. Benefitting from the optimized interfacial electrical contact and photoresponse, the perovskite/In-BTC heterojunction-based PSCs yielded enhanced PCE (19.63 ± 1.24%), outperforming the pristine devices (18.19 ± 1.33%). Besides, over 80% of the initial PCE could be maintained for the In-BTC-modified PSCs after being stored in high-humidity environment for 12 days without encapsulation, exceeding the 35.4% left to pristine devices. These results demonstrate that it is a facile and effective strategy to fabricate efficient and stable PSCs by employing perovskite/In-BTC heterojunction as the light-harvesting layer.

## Electronic supplementary material

Below is the link to the electronic supplementary material.

Supplementary material 1 (PDF 1308 kb)
