# Supplementary material for: Heterojunction Incorporating Perovskite and Microporous Metal–Organic Framework Nanocrystals for Efficient and Stable Solar Cells
Source: Nanomicro Lett. 2020 Mar 28;12:80. doi: 10.1007/s40820-020-00417-1 (PMC7770709; doi:10.1007/s40820-020-00417-1)
Supplement: Supplementary file 1 — Supplementary material 1 (PDF 1308 kb) [file 40820_2020_417_MOESM1_ESM.pdf]

Supporting Information for

Heterojunction Incorporating Perovskite and Microporous Metal-Organic Framework Nanocrystals for Efficient and Stable Solar Cells

Xuesong Zhou[1], Lele Qiu[1], Ruiqing Fan[1, *], Jian Zhang[1], Sue Hao[1], Yulin Yang[1, *]

[1]MIIT Key Laboratory of Critical Materials Technology for New Energy Conversion and Storage, School of Chemistry and Chemical Engineering, Harbin Institute of Technology, Harbin 150001, People's Republic of China

*Corresponding authors. E−mail: fanruiqing@hit.edu.cn (Ruiqing Fan), ylyang@hit.edu.cn (Yulin Yang)

Supplementary Figures

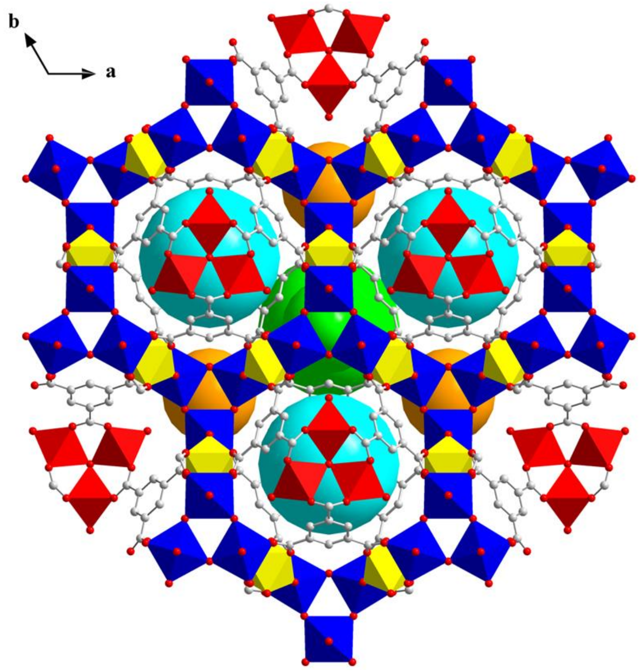

Fig. S1 Indium octahedral representation of In-BTC 3D framework along the *c*-axis, showing the 18-membered hexagonal ring-based infinite 2D layers (In2 and In3 octahedrons: yellow and blue, respectively) linked to the isolated μ_3-oxo-centred trinuclear units (In1 octahedron: red) through the $[btc]^{3-}$ ligands. Spheres are located into each cavity for a better clarity of the volume.

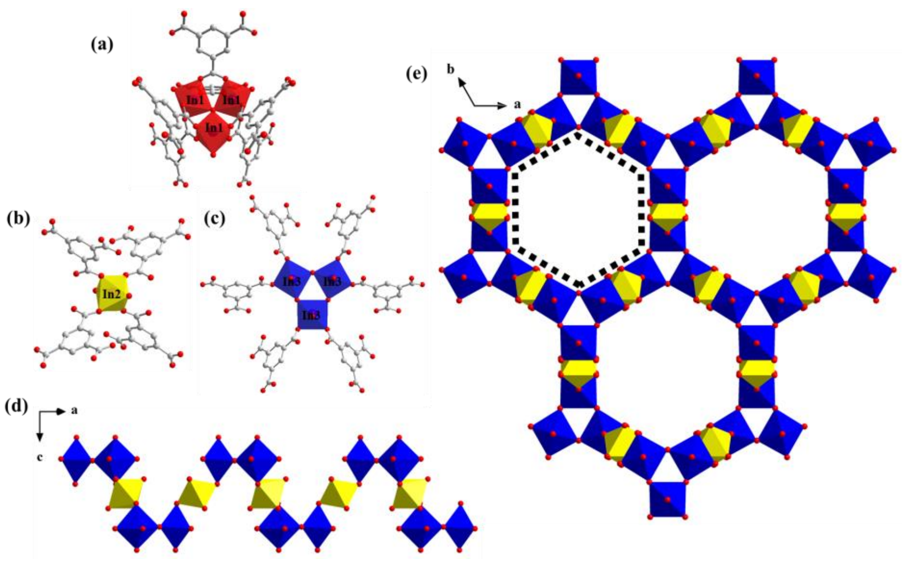

Fig. S2 Coordination environment of (**a**) In1, (**b**) In2, and (**c**) In3. Polyhedral representation of infinite 2D layers on *ab* plane based on corner-shared In2 and In3, viewed along the (**d**) *b* and (**e**) *c* axes

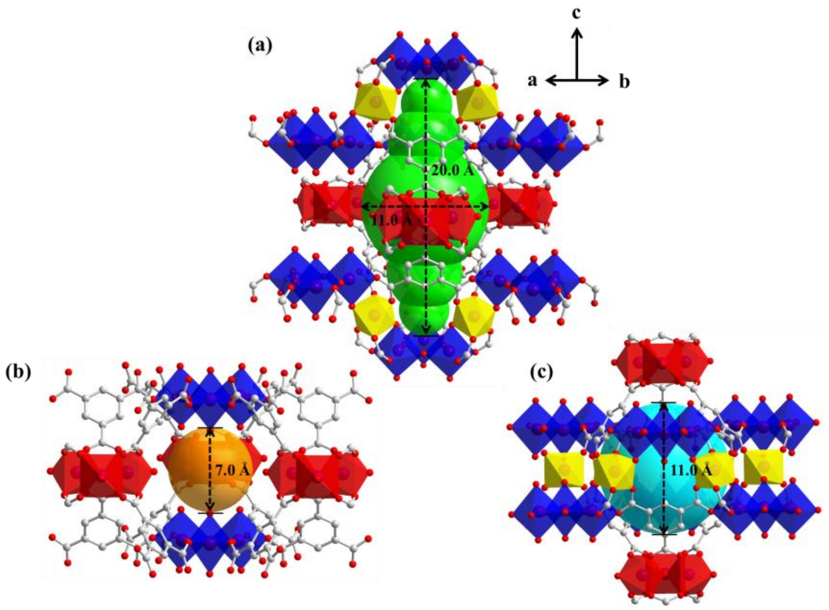

Fig. S3 Representation of three different types of cavities observed in In-BTC structure. Spheres are located into each cavity for a better clarity of the volume. (**a**) The first cavity is delimited by three trinuclear units (In1, red), two hood-shaped units composed of In2/In3-centered octahedrons (yellow and blue), and six $[btc]^{3-}$ ligands. (**b**) The second one is situated between three trinuclear units on the *ab* plane and two trimeric units (six In3-centered octahedrons) along the *c* axis. (**c**) The third one is delimited by two trinuclear units along the *c* axis connected to the 18-membered ring on the *ab* plane *via* six $[btc]^{3-}$ ligands.

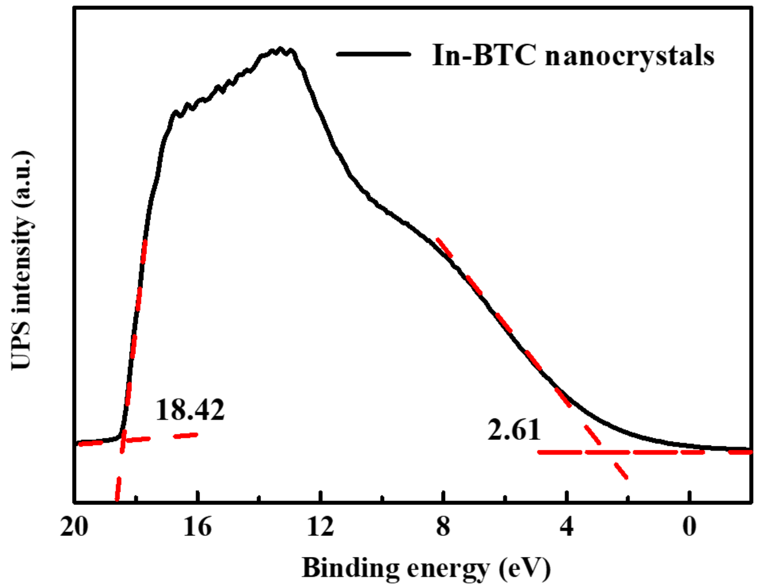

Fig. S4 UPS spectra of In-BTC nanocrystals

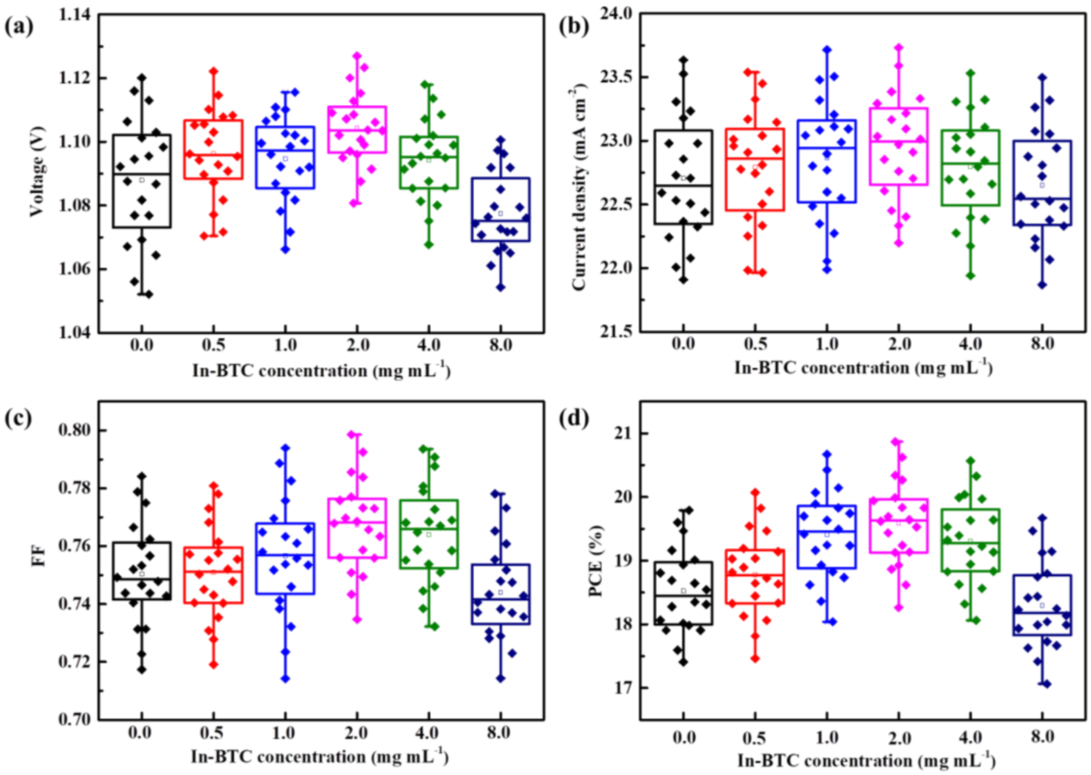

Fig. S5 Statistical distribution of the (**a**) V_{oc}, (**b**) J_{sc}, (**c**) *FF*, and (**d**) PCE for PSCs employing perovskite/In-BTC heterojunction with different addition concentrations of In-BTC nanocrystals

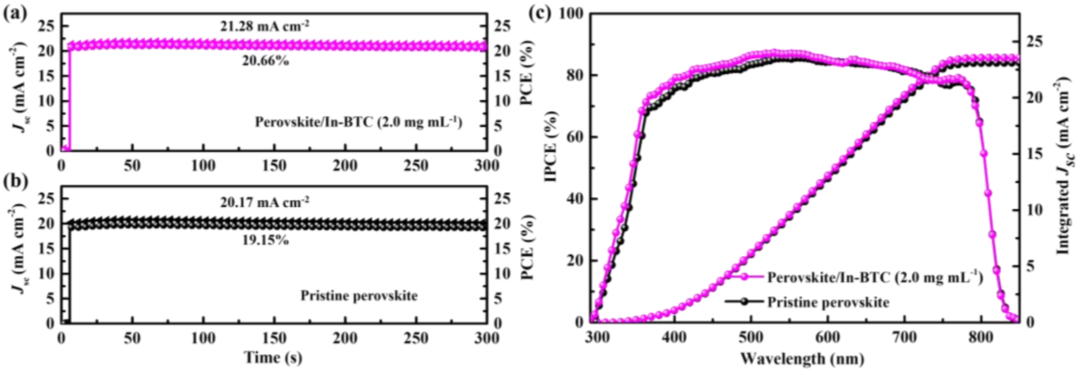

Fig. S6 Steady-state photo-current output at the maximum power point and corresponding power output for PSCs with the (**a**) optimal perovskite/In-BTC heterojunction (2.0 mg mL^{-1}) or (**b**) pristine perovskite. (**c**) IPCE spectra of the pristine and In-BTC-modified devices, and corresponding integrated J_{sc}

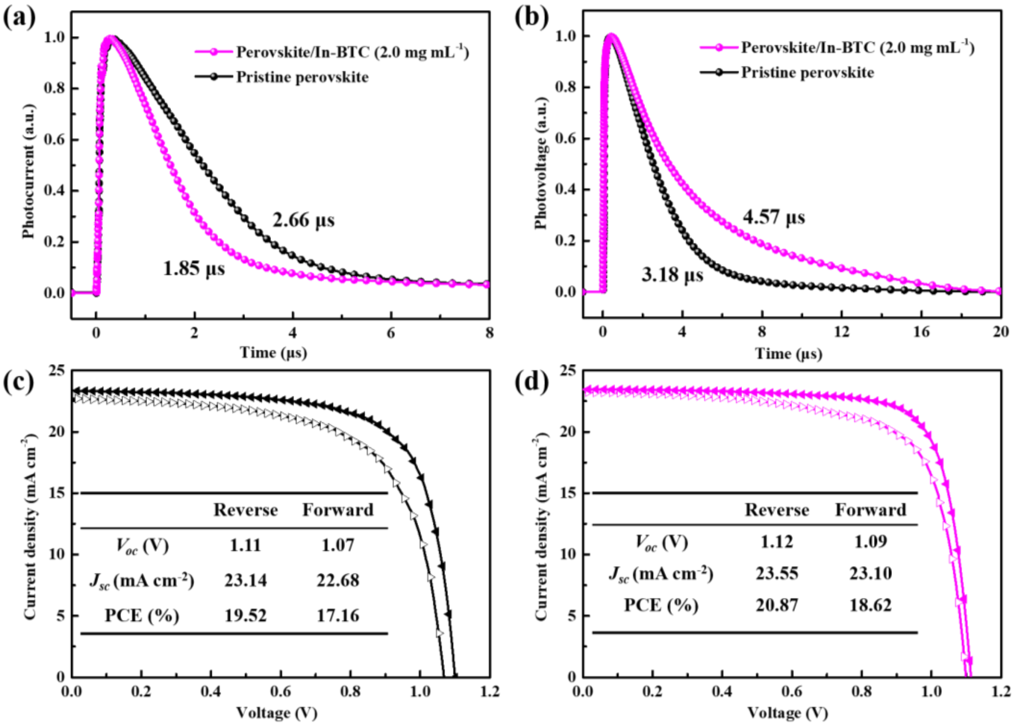

Fig. S7 (**a**) Transient photocurrent decay and (**b**) transient photovoltage decay for PSCs with pristine and In-BTC-modified perovskite films. The *J*-*V* curves of devices with (**c**) pristine and (**d**) In-BTC-modified perovskite films in the forward and reverse scanning directions

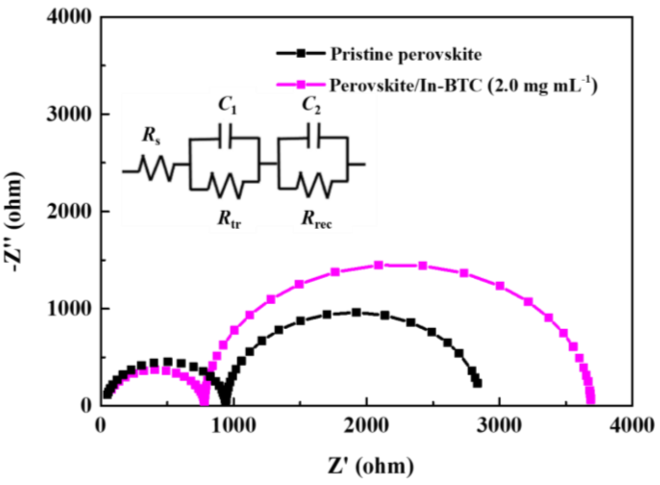

Fig. S8 EIS measurement spectra for the PSCs with pristine perovskite or perovskite/In-BTC heterojunction (2.0 mg mL^{-1}). (The inset is the equivalent electrical circuit for fitting the EIS data)

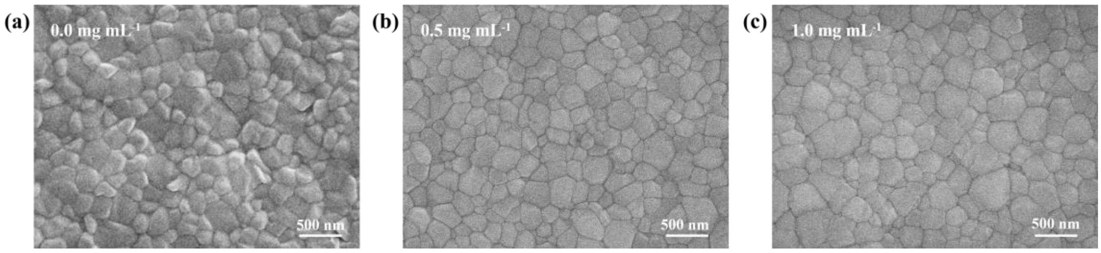

Fig. S9 SEM images of (**a**) pristine perovskite thin films and (**b, c**) perovskite/In-BTC heterojunction films with different addition concentrations of In-BTC nanocrystals

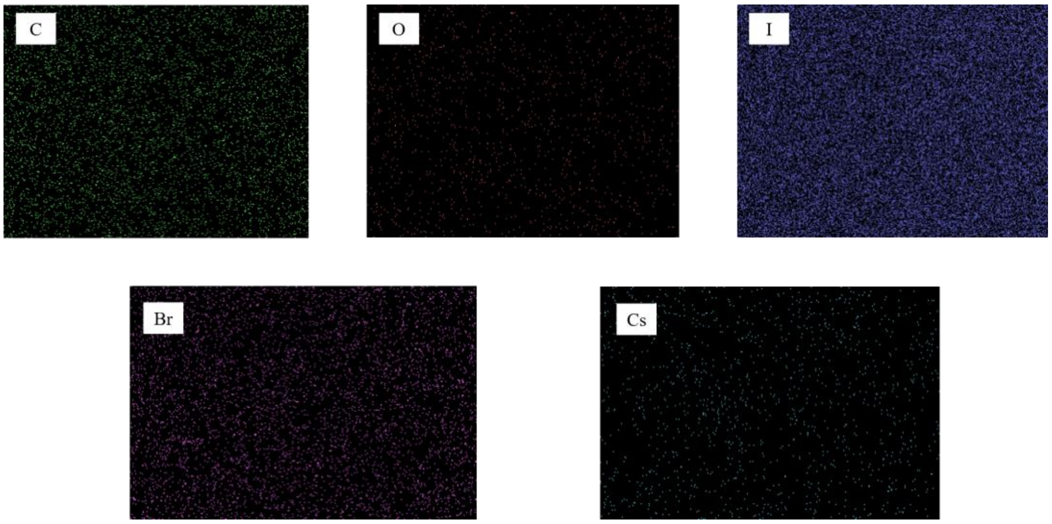

Fig. S10 SEM-EDS images of optimal perovskite/In-BTC heterojunction film (2.0 mg mL^{-1})

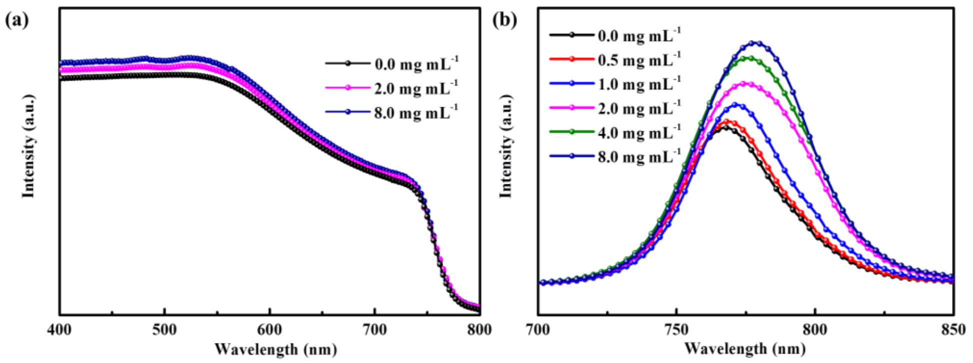

Fig. S11 (**a**) UV-vis absorption and (**b**) PL emission spectra of perovskite/In-BTC heterojunction films with different addition concentrations of In-BTC nanocrystals. (The UV-vis absorption curves corresponding 0.5, 1.0, and 4.0 mg mL^{-1} are selectively hidden for a clearer resolution)

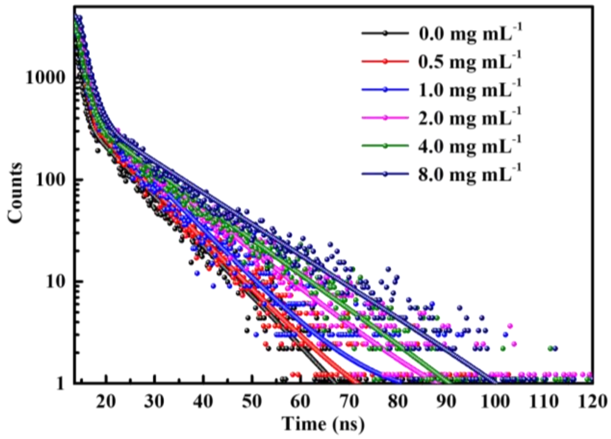

Fig. S12 Time-resolved photoluminescence (TRPL) spectra of perovskite/In-BTC heterojunction films with different addition concentrations of In-BTC nanocrystals

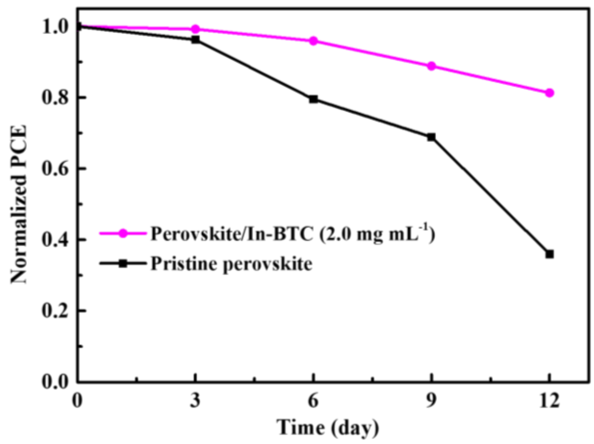

Fig. S13 Normalized PCE of corresponding devices for different storage time in air (25 °C and RH: ~65%)
